# Lactation in domestic carnivores

**DOI:** 10.1093/af/vfad027

**Published:** 2023-06-14

**Authors:** Sylvie Chastant

**Affiliations:** NeoCare, ENVT, Université de Toulouse, 23 Chemin des Capelles, Toulouse, France

**Keywords:** cat, colostrum—milk, composition, dog pseudopregnancy

**Implications**

- Data on the dog and cat milk composition allow for the evaluation of the composition of industrial milks (of frequent use in these species).
- Available data on canine and feline milk were obtained on very limited numbers of females.
- Quantities of colostrum and milk produced per female and variations factors of production and composition are poorly understood, making the improvement of lactation in these species difficult.
- Colostrum is not of markedly greater energetic concentration than milk, but has a crucial role in the acquisition of passive immunity by newborns, since puppies and kittens are born agammaglobulinemic.
- Quantity and quality of mammary secretions vary according to mammary gland but no dam-side test allows to target suckling of the secretions of the greatest quality.

## Introduction

Lactation in dogs and cats received limited interest until recently, probably due to the absence of interest in carnivore milk and young growth for human nutrition. The robustness of knowledge available about lactation in these species is hindered by the limited number of publications, small number of dams involved, together with the obsolescence of the analytic methods used. Nevertheless, research on canine and feline colostrum was recently stimulated by the demonstration of the importance of passive immune transfer in neonatal survival (Chastant and Mila, 2019), because of an endothelial placenta, maternal immunoglobulins cannot reach the fetal bloodstream during pregnancy, making puppies and kittens totally dependent on colostrum ingestion for the acquisition of their immunity over the neonatal period. Interest in canine and feline lactation is also growing in line with recent data obtained in various species demonstrating the key role of neonatal and pediatric nutrition on the short- and long-term health of the individual (Vickers, 2014; Acevedo et al., 2021).

### Endocrine context of lactation

Lactation is induced by a sharp decline in blood progesterone concentration at the time of parturition. Canine lactation can begin as early as 2 weeks before parturition but can also be belayed to 2 to 3 days after parturition. It peaks at 3 weeks postpartum and lasts around 3 months (unless puppies are conventionally weaned around 6 weeks of age). In dogs, lactation occurs during the anestrus phase (no ovulation, no luteal phase). Feline lactation lasts 3 to 4 months (depending on the presence of kittens, most often weaned later than puppies, around 10 weeks) and is different than bitches, female cats can cycle, ovulate, and become pregnant during lactation.

### Anatomy of lactation

The bitch classically presents 10 mammary glands (8 to 12 depending on the individual), thoracic to inguinal whereas, female cats only have eight glands (four pairs). Lacteal ducts do not merge into a cisterna as in ruminants, but open directly at the surface of the teat, through 7 to 20 distinct canals and openings. Manual milking thus requires gentle massage on mammary tissue rather than pressure on the nipple. The diameter of the alveoli doubles from parturition to the third week of lactation, with the number of alveoli per lobule remaining high over the first week of lactation and the first 40 days of lactation (Orfanou et al., 2010).

### Colostral phase

The colostral phase is defined in carnivores as the 2 first days after parturition, based on immunoglobulin (Ig) G concentration dramatic decline in mammary secretions after parturition (−50% over the first 24 h after parturition—Chastant et Mila, 2019 in dog; Claus et al., 2006 in cat). The actual quantity of colostrum produced by bitches and queens is unknown. If (over)estimated from milk production measured during the first week post-partum, daily colostrum production can be calculated as 2.7% (1–6%) and 4.1% (1–8%) of the dam’s body weight in bitches and in queens, respectively (Meyer and Zentek, 2005; Dobenecker et al., 1998), compared with around 1% for dairy cows (10 kg colostrum over the first-day post-partum for an 800-kg female).

Table 1 summarizes the composition of dog and cat colostrum in comparison with their mature milk and bovine colostrum/milk. Part of the large variability observed might be related to the different analytic methods used, with the one used in the more ancient publications probably lacking accuracy and/or specificity. The energetic value of carnivore colostrum, not markedly greater than that of mature milk, is provided at around 50% by proteins and 40% by lipids (Chastant-Maillard et al., 2017).

**Table 1.** Mean composition of mammary secretions in carnivores species (compared with bovine)

Parameter	Unit	Canine colostrum	Canine milk	Feline colostrum	Feline milk	Bovine colostrum	Bovine milk
Dry matter	%	20–25	21–26	25	25–30	24–28	12.9
Proteins	g/100g milk	8–14	7–8	4–8	6–9	14–16	3.1–3.2
Lipids	g/100g milk	10–13	8–12	2–13	5–13	6–7	3.6–4.0
Lactose	g/L	16–34	30–50	30–40	40	20–30	47–51
Energy	kcal/L	1,300–1,800	1,500	1,300	1,200	1,300	640
IgG	g/L—mean/dam	20–40	2–3	50–70	2–5	50	0.4–0.9
IgA	g/L—mean/dam	10–25	8–10	1.4	0.3	3.2–6.2	0.05
Calcium	g/kg	1.3	2	0.4	2	2.6–4.7	1.2–1.3
Osmolarity	mOsm		569		329		308

Since carnivore newborns are born agammaglobulinemic, immunoglobulin provision by colostrum is crucial for their health and survival. The intestinal barrier remains permeable to such macromolecules only during 12–24 h (Casal et al., 1996; Chastant et Mila, 2019). IgG is the major class present in the colostrum (60–75% of total immunoglobulins in dog colostrum, 98% in cat colostrum; Claus et al., 2006; Chastant-Maillard et al., 2017). IgG concentrations seem greater in feline (mean 62 g/L) than in canine (mean 20 g/L) colostrum, but in both species, IgG colostral concentration is highly variable between dams (3.1–68.8 g/L among 139 bitches; 18.6–136.0 g/L among 65 female cats). IgG concentration is 2 to 3 times greater in dog colostrum than in maternal serum (between 0.9 and 6.3 times depending on the dam), and 4.5 ± 1.9 times greater in the feline species (Claus et al., 2006), without any correlation between colostral and maternal serum IgG concentrations. The repeatability of colostrum immune quality along with successive lactations for one given bitch has not been explored. Nevertheless, IgG colostrum concentration does not seem to be a limiting factor of the passive immune transfer, rather seems to be limited by the quantity of colostrum ingested and the time elapsed between birth and ingestion (Chastant and Mila, 2019). Finally, the immune quality (evaluated through Ig G concentration) and energetic value of colostrum are not correlated (Chastant-Maillard et al., 2017).

Colostrum is not only responsible for immunity and energy provision but also for organic growth and differentiation. Many bioactive compounds, growth factors, and hormones (such as insulin, cortisol, and thyroxin) have been identified in canine colostrum. Two enzymes, gamma-glutamyl transferase and alkaline phosphatase, exhibit high concentrations, respectively, 100 times and 10 times more than in maternal serum (Center et al., 1991). Since they are essentially absent from the circulating blood at birth, their detection of these enzymes in a puppy’s serum confirms ingestion of colostrum (although the enzyme levels do not correlate to the IgG concentration). Colostrum also contains cellular elements, namely, polymorphonuclear neutrophils and exosomes (those last carrying proteins involved in angiogenesis, metabolism, cell signaling, and probably in fat reserves development) (Rossi et al., 2021; Demattio et al., 2022). Colostrum also carries a specific microbiota, which raised a recent interest for its potential impact on gut puppy’s bacterial colonization and thus health (Kajdic et al., 2021; Del Carro et al., 2022).

### Milk phase

The quantity of milk produced daily is only estimated through only a few studies, with large variations in estimates between studies. In both species, milk production varies with the stage of lactation (with a peak around 3 weeks post-partum) and markedly increases with litter size (Dobenecker et al., 1998; Meyers and Zentek, 2005). In the female cat, for the first week of lactation, Dobenecker et al. (1998) estimated daily milk production by weighing kittens before and after suckling to 1% (for litters of one to two kittens) to 3.1% (six kittens) of maternal body weight. At 2 to 4 weeks of lactation, daily milk production increased, respectively, to 1.3% and 5.9% of dam body weight for the same litter sizes. Similarly, in the dog, milk production is 3 times more important in bitches with large litter sizes (> 6 puppies) compared with small (< 4 puppies). Daily milk production is 1.7–4.4% of maternal body weight in the first week of lactation and increases to 2.8–6.6% in the third to fourth week of lactation (Meyer and Zentek, 2005). Oftedal (1984) estimated milk production to be 7.6–8.3% of maternal body weight at days 19 to 26 of lactation. For comparison, a cow exports around 4% of her body weight as milk.

The gross composition of dog and cat milk compared with bovine milk is provided in Table 1. As with bovine milk, milk composition is constant during a suckling/milking session (Jacobsen et al., 2004) in cat. Compared with bovine milk, dry matter percentage in canine and feline milk is 2-fold greater, together with the energetic value. Similar to bovine milk, the predominant milk protein class in dog milk is casein (whey/casein ratio 30:70). However, in cat milk, whey proteins dominate (whey/casein ratio 60:40; Adkins et al., 1997, 2001). Data about specific elements are also available, such as minerals and trace elements (Keen et al., 1982; Adkins et al., 1997, 2001; Dobenecker et al., 1998), amino acids (Adkins et al., 1997, 2001; Heinze et al., 2014), fatty acids (Jacobsen et al., 2004; Heinze et al., 2014), oligosaccharides (Macias Rostrami et al., 2014), and nucleotides (Tonini et al., 2010).

Milk also contains hormones (progesterone and relaxin), antimicrobial substances (lactoferrin and lysozyme), growth factors (epidermal growth factor, transforming growth factor beta, insulin-like growth factor 1), and bacteria (Steinetz et al., 2008; Vasiu et al., 2021). Milk antibodies (mainly IgA secreted in the mammary tissue) contribute to the newborn local intestinal health, acting in the lumen after the intestinal barrier closure.

### Physiological variations

#### Modifications of milk composition with lactation stage.

**Figure 1.**
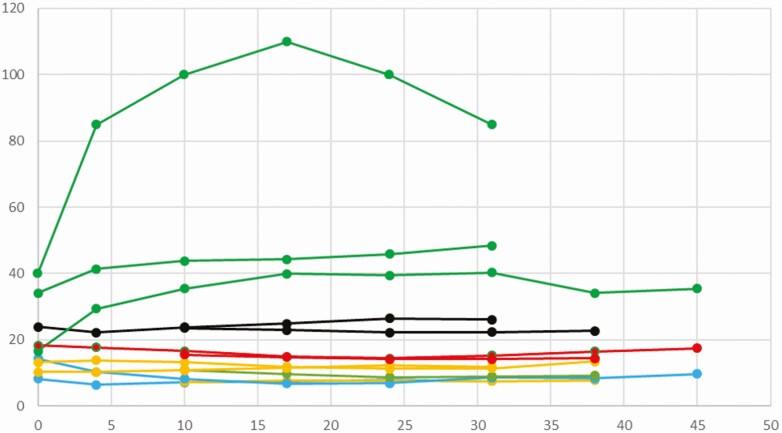
Changes in mammary secretions along with lactation. Canine species. (From Oftedal, 1984, *n* = 5 bitches; Adkins et al., 2001, *n* = 10; Macias Rostrami et al., 2014, *n* = 7; Dokoupilova et al., 2016, *n* = 9.) Green: lactose (g/l); black: dry matter (g/100 g); red: energy (kcal/cl); orange: lipids (g/100 g); blue: proteins (g/100 g).

**Figure 2.**
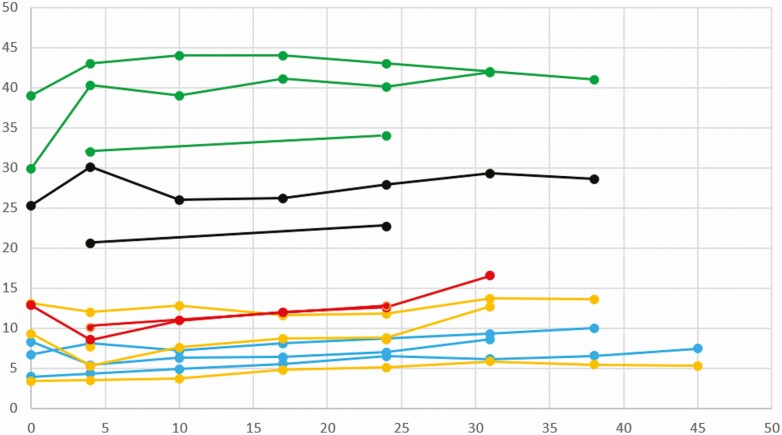
Changes in mammary secretions along with lactation. Feline species. (From Keen et al., 1982, *n* = 7 female cats; Adkins et al., 1997, *n* = 12; Dobenecker et al., 1998, *n* = 6; Jacobsen et al., 2004*n* = 11.) Green: lactose (g/L); black: dry matter (g/100 g); red: energy (kcal/cl); orange: lipids (g/100 g); blue: proteins (g/100 g).

Very few publications performed a follow-up of milk composition along lactation. Figure 1 (dog, *n* = 4 studies, 31 bitches in total) and Figure 2 (cat; *n* = 4 studies, 54 females) report data from publications before 1982 having assayed samples repeatedly on the same females. From this very limited dataset, some trends can be described. In dogs, lactose concentration increases over the first week of lactation, in parallel to a decrease in protein percentage. Lipids and energetic values remain constant over lactation (Figure 1). In the feline species, discrepancies appear in the first week. Carbohydrates percentage and dry matter proportion remain constant, whereas protein and lipid percentages increase progressively during lactation (Figure 2).

#### Interindividual variations.

In contrast to data in bovine, in which the effect of parity, age, and breed on colostrum/milk production are well described, interindividual variations are difficult to document in canine and feline species due to the limited number of animals included in each experiment. Only the impact of litter size is partially described (see above). Moreover, the amplitude of interindividual variation differs among nutritional/immune parameters. For example, one study collected colostrum from 81 bitches reported a ratio between the greatest and the least concentrations obtained for one animal of 40 for IgG, 15 for lipids, and only 2 for protein and sugar concentration (NeoCare, unpublished data).

#### Functional differences between mammary glands.

The functional equivalence of the different mammary glands can be questioned, both from the dam’s side and from the newborn’s side. As evaluated in 21 bitches, the intrabitch coefficient of variation between mammary pairs is high for fat percentage (26% ± 12%) and IgG concentration (28% ± 28%). For one given bitch, the greatest and the least IgG concentrations as assayed by mammary pairs differ in average by a factor of 5.9 (Chastant-Maillard et al., 2017). Since the position of the mammary gland pair secreting the colostrum with the greatest IgG concentration is not constant among bitches, no practical recommendation can be drawn for dog breeders to encourage the suckling of a specific pair of mammary glands to optimize passive immune transfer. The suckling behavior of puppies (5 ± 2 teats suckled over the first 12 h of life) contributes to neutralizing these differences in colostrum immune quality. In the female cat, proteins and lipids concentration does not differ between cranial and caudal mammary glands, and the superiority of caudal mammary glands for lactose concentration, despite being statistically significant, only accounts for a 5% variation (Jacobsen et al., 2004).

Mammary glands are also not equivalently suckled by newborns. As soon as 12 h of life, kittens develop a preference for the posterior nipples; 40% of the suckling time is spent on posterior nipples versus 5% and 20% on cranial (Hudson et al., 2009). Puppies spend 30% of the suckling time on mammary gland 5 (inguinal) versus 17% for others over the first 24 h of life (Chastant and Mila, 2019).

### Sanitary risks associated with lactation

Colostrum and milk may contain potential pathogens for the newborns, such as viruses (namely, FeLV and FIV), parasites *(Toxocara canis* and *cati*, *Neospora caninum*), and pathogenic bacteria. They can also mediate xenobiotics administered to the dam, meaning that any drug given to a lactating dam has to be evaluated in regard to its lactogen transmission and its innocuity for newborns. In the feline species, colostral antibodies may be responsible for neonatal isoerythrolysis in blood group A kittens born from a B female (Giger and Casal, 1997). From the maternal side, lactation exposes the dam to mastitis and eclampsia (Gonzalez, 2018; Vasiu et al., 2021).

### Lactation without parturition in the bitch

Physiologically, lactation is induced by the sharp decline in concentrations of progesterone and an increase in prolactin in blood at the end of pregnancy. Such a hormonal context also takes place at the end of the luteal phase, making the bitch able to produce mammary secretions in the absence of parturition (Concannon, 2011). This phenomenon, called pseudocyesis or overt pseudopregnancy, is considered as non-pathological, since it is shared with other canids allowing allomaternal care of puppies of the pack. Subordinate non-mothers usually lactate the dominant female’s puppies (Gobello, 2021) and interestingly, concentrations in immunoglobulins (IgG, IgA) are equivalent in pseudopregnancy mammary secretions than in colostrum (Chastant and Mila, 2019). In cats, pseudopregnancy can also develop after spontaneous ovulation or ovulation induced by an infertile coitus but is not associated with lactation in this species.

### Manipulation of lactation

Before whelping, diet modifications ensure optimal quantitative milk production. Interestingly, female dogs and cats differ in terms of source of nutrients for lactation; from ingested food in the bitch versus adipose tissue deposited during pregnancy in the cat. As a consequence, dietary energy is increased prepartum beginning at mating in cats but only from the sixth week of pregnancy onwards in the bitch (+150% of maintenance level) (Greco, 2008). Postpartum nutrition has also a major impact on the quantity of milk produced, together with access to water (Jacobsen et al., 2004; Fontaine, 2012).

In addition to food quality, ingestion may limit milk production. In practice, special attention is paid to postpartum pain, environmental stress, and maternal behavior, such as some dams with overdeveloped maternal instincts refusing to leave their litter to eat and drink.

Milk production can also be pharmaceutically modulated. Metoclopramid, an antidopaminergic drug usually used as antiemetic, stimulates prolactin secretion, and thus helps early lactation induction when administered during the first days postpartum. Conversely, dopaminergic antiprolactinic drugs such as cabergolin or metergolin, allow dry-off, in cases of newborn deaths or mastitis. From a qualitative aspect, mammary secretions can be marginally modified through maternal diet supplementation with prebiotics and probiotics or fatty acids (Bauer et al., 2004; Adogony et al., 2007; Alonge et al., 2020). Vaccination booster shortly before mating will enrich colostrum in antibodies targeting neonatal and pediatric pathogens (e.g., feline and canine herpesvirus and parvovirus).

## Conclusion

Compared with ruminant species, basic knowledge is lacking concerning colostrum and milk in the dog and the cat, beginning with quantitative data on daily production. The limited number of studies and females from which available data were obtained make them fragile, requiring confirmation (or not) by further observations. Precise composition remains to be confirmed and factors (both maternal and environmental) affecting colostrum/milk quantity and quality also need deep and detailed exploration. Colostrum and milk remain elegant and potent tools to improve neonatal and pediatric health; a novel area of research is fully open to explore the strategies to apply during or even before pregnancy on dams to modulate chemical or microbiota composition of colostrum and milk to the benefit of puppies and kittens health.

## About the Author

Professor Sylvie Chastant (DVM, MSc, PhD, Hab, DiplECAR) teaches Animal Reproduction at Toulouse National Veterinary School (Toulouse, France). She is the vice-president of the European College of Animal Reproduction (ECAR) and member of the French Veterinary Academy. Her main interests are teaching and clinical/applied research, with a multispecies comparative approach. She created a lab dedicated to neonatalogy and reproduction in carnivores, aiming to improve health of puppies, kittens, and their dams, together with sustainability of kennels and catteries.
